# Comparison of machine learning models for bradykinesia assessment using digital health technology

**DOI:** 10.3389/fdgth.2026.1854818

**Published:** 2026-08-21

**Authors:** Matthew D. Czech, Yu Deng, Josh Cosman, Michelle Crouthamel, Sheng Zhong, Li Wang, E. Ray Dorsey, Jamie L. Adams, Jie Shen

**Affiliations:** 1 Data and Statistical Sciences, AbbVie, North Chicago, IL, United States; 2 Department of Neurology, University of Rochester Medical Center, Rochester, NY, United States

**Keywords:** bradykinesia, deep learning, digital health, machine learning, Parkinson’s disease, wearables

## Abstract

**Background:**

Bradykinesia, a primary symptom of Parkinson’s disease, significantly impacts patients’ quality of life. Traditional assessments like the MDS-UPDRS require in-person evaluation and can be subjective, resulting in patient burden and variability.

**Methods:**

This study investigates digital health technologies and machine learning (ML) models, specifically XGBoost and Convolutional Neural Networks (CNNs), to improve objectivity and precision in bradykinesia quantification. Using data from the 12-month WATCH-PD study in early, untreated Parkinson’s disease, models were evaluated against MDS-UPDRS items (3.6 pronation-supination and 3.7 toe tapping) for cross-sectional accuracy and longitudinal sensitivity.

**Results:**

Results show that the class-balanced XGBoost was the best-performing model on both tasks, outperforming a stratified-random baseline and all CNN variants (cross-entropy, focal loss, and self-attention) across every imbalance-aware metric: macro-F1 0.47 and 0.40, balanced accuracy 0.47 and 0.42, quadratic weighted kappa 0.58 and 0.47, and accuracy 0.60 and 0.53 for pronation-supination and toe tapping, respectively, with the clearest gains on the under-represented higher-severity classes. A secondary longitudinal analysis found limited sensitivity to 12-month change across the ML models.

**Discussion:**

Overall, machine-learning models, led by the class-balanced XGBoost, show promise in accurately predicting item-level MDS-UPDRS bradykinesia scores from wearable sensor data. Our results also highlight the importance of aligning feature selection and model architecture to intended clinical endpoints.

## 1 Introduction

Bradykinesia, characterized by slowness of movement, is a hallmark symptom of Parkinson’s disease and significantly impacts the quality of life for those affected [1]. Accurate quantification of bradykinesia plays a crucial role in assessing disease severity, monitoring progression, and evaluating treatment efficacy. Traditionally, clinical assessments such as the Movement Disorder Society-sponsored revision of the Unified Parkinson’s Disease Rating Scale (MDS-UPDRS) have been employed to quantify bradykinesia [2]. However, these assessments are often subjective and may suffer from inter-rater variability.

In recent years, technological advancements have paved the way for utilizing digital health technologies (DHTs) such as wearables and computational methods to enhance the objectivity and precision of motor function evaluation, including bradykinesia assessment [3–6]. Heuristic methods that leverage signal processing techniques and advanced knowledge of functional biomechanics have been widely used to translate time series inertial measurement unit (IMU) data into clinically understandable features [7, 8]. In contrast, machine learning (ML) algorithms have gained significant attention for their ability to analyze complex data and identify hidden patterns that may not be apparent through heuristic techniques [9]. For instance, various ML approaches have demonstrated promising results in domains such as gait analysis, where in some cases they have outperformed traditional heuristic methods [10].

Despite these advances, there remains a gap in understanding which computational strategies might be most effective for quantifying specific motor symptoms like bradykinesia. The selection of an appropriate algorithm can be crucial as different methods may offer complementary strengths, and therefore, understanding their comparative performances is imperative for the development of robust assessment tools. In this study, we aim to address this gap by training different types of ML models—specifically, gradient-boosted decision trees (XGBoost) and convolutional neural networks (CNN)—and evaluating performance against relevant MDS-UPDRS items, specifically item 3.6 (pronation-supination movement of hands) and item 3.7 (toe tapping), as well as their ability to track disease progression over time in a longitudinal observational study [11, 12]. Specifically, we compare their performance in: (i) cross-sectional classification against item-level MDS-UPDRS ratings, (ii) correlations between model outputs and total motor MDS-UPDRS scores, and (iii) effect-size analyses capturing change from baseline to 12 months. By doing so, we seek to determine the effectiveness of various algorithms in capturing bradykinetic movements and to explore whether ML methods hold additional advantages over heuristic approaches.

Compared to prior work [e.g., [5, 13]], which primarily focused on developing single-method heuristic or ML models for bradykinesia, our study introduces a comparative ML evaluation framework using a longitudinal dataset collected over 12 months. This framework integrates both cross-sectional and longitudinal perspectives, enabling hybrid insights into the strengths and limitations of different computational approaches. We also benchmark ML outputs against established clinical endpoints to systematically examine how algorithm choice impacts both snapshot mapping and progression tracking, offering methodological guidance not addressed in earlier studies. This exploration not only contributes to the ongoing discourse on computational methods for bradykinesia quantification but also provides valuable insights that may guide future research and clinical practice. Ultimately, our goal is to enhance the accuracy and reliability of bradykinesia assessment in clinical practice and advance methodological approaches within the research community.

## 2 Materials and methods

### 2.1 Participants and dataset

This study analyzed data from the WATCH-PD trial, a 12-month multicenter observational study of early Parkinson’s disease [11, 12]. The cohort included 82 individuals with early, untreated PD (ePD) and 50 age-matched healthy controls. Participants completed standardized motor assessments at baseline and regular follow-up clinic visits (approximately at months 1, 3, 6, 9, and 12) as part of the study design. These assessments mapped directly onto MDS-UPDRS Part III tasks, including upper-limb pronation–supination and lower-limb toe tapping exercises, to objectively measure bradykinesia. During each in-clinic assessment, subjects wore six inertial sensors (research-grade APDM Opal devices), four placed on the limbs and two on the torso [14]. The inertial sensors (each containing tri-axial accelerometers and gyroscopes) continuously record kinematic data throughout the performance of each motor task, while trained clinicians contemporaneously score each assessment. For bilateral tasks (e.g., left and right hand or foot movements), each side’s performance was treated as an independent sample labeled with its corresponding MDS-UPDRS item score. This yielded an augmented dataset of time-series signals paired with clinician-rated bradykinesia scores, collected over multiple time points for both PD and control participants. After excluding visits with missing clinical data or technical problems with data capture, a total of 128 participants and over 1,100 task recordings were available for analysis in this study (covering both pronation–supination and toe tapping tasks at the subject-visit level).

### 2.2 Modeling approach

We implemented two complementary ML frameworks to predict task 3.6 (pronation-supination movement of hands) and 3.7 (toe tapping) MDS-UPDRS bradykinesia scores from the wearable sensor data: an ensemble gradient-boosted decision tree model (XGBoost) and a one dimensional (1D) convolutional neural network (CNN). XGBoost was selected for its strong performance across diverse tasks and ability to model complex nonlinear relationships. The 1D CNN was used because its convolutional kernels capture temporal patterns and can directly model correlations in raw time-series data. The modeling workflow was structured to include common preprocessing steps for both models, followed by distinct feature engineering steps specific to each approach, and then respective training procedures for each model.

These input strategies reflect the models’ architectures: as a tree ensemble, XGBoost treats features as independent and cannot learn directly from raw sequential data, so each signal segment is summarized by engineered time- and frequency-domain features; the CNN instead learns hierarchical temporal features directly from the full raw sequence through its convolutional and pooling layers. The corresponding segmentation is described next.

### 2.3 Data preprocessing and segmentation

All sensor data underwent preprocessing to isolate the segments corresponding to active task performance. Each raw trial recording was segmented to identify the interval of purposeful movement for the pronation–supination or toe tapping task, as described in our previous work [13]. In practice, a rolling-window variance computed on the raw device orientation data was used to extract the roughly 10 s window of each task where movement was most prominent. This step removed noisy or inactive portions at the beginning and end of trials, ensuring that subsequent analysis focused on high-quality movement signals. Visual verification was used to confirm segmented datasets. For the XGBoost pipeline, each 10 s task segment was further divided into overlapping 3 s windows (with 1 s overlap) to extract comprehensive summary statistics and capture temporal patterns within each window. We chose 3s window with 1s overlapping because it provides relatively high temporal resolution with more granular capture of movement patterns and transitions. The 1s overlap ensures continuity without excessive redundancy. This yielded five partially overlapping sub-segments per trial, each assumed to contain representative movement patterns of the task. These sub-segments were then concatenated to form a unified feature set for XGBoost model prediction. Recordings whose active-movement segment was too short to form these windows (two toe tapping recordings with <4 s of usable data) were excluded from the XGBoost feature set. By contrast, the CNN pipeline utilized the entire 10 s segmented sequence per task as the model input, rather than smaller windows, since the CNN could learn features directly from the continuous time-series data. In both pipelines, we used only the accelerometer and gyroscope signals from the wearable sensors (sampled at 128 Hz) as model inputs. The sensor signals were normalized and synchronized across axes and devices as needed. Both CNN and XGBoost rely on identical sensor-only inputs, ensuring a fair model comparison. Models were trained and evaluated based on four MDS-UPDRS score classes: 0, 1, 2, and 3 (combined with 4 due to lack of sample size).

### 2.4 XGBoost model

For the gradient-boosted tree approach, we engineered a comprehensive set of time-domain and frequency-domain features from each 3 s sensor window. These hand-crafted features were designed to capture relevant characteristics of bradykinetic movements—for example, signal amplitude variability, peak angular velocities, frequency content of repetitive movements, and signal entropy, among others (a complete feature list is provided in the Supplementary Material Table S4). Window-level features within each task segment were concatenated to generate unified feature vectors. The XGBoost model was then trained to classify the MDS-UPDRS score using these features as inputs. Here, we define a task segment as 10 s of sensor data collected during a single MDS-UPDRS task (pronation-supination or toe tapping) performed on one side (left or right) during a specific visit. The XGBoost classifier predicts one out of the four classes (0, 1, 2, and 3) using the sensor on the tested limb. To address class imbalance, we additionally trained a variant with class-balanced sample weights (weights inversely proportional to class frequency). Key hyperparameters (e.g., the number of trees in the ensemble, maximum tree depth, learning rate, subsampling fraction, and feature subsampling ratio, see Table 1) were tuned via grid search on the training data using 5×5 nested cross-validation. Hyper-parameters such as number of trees, maximum tree depth, subsampling fraction, and feature subsampling ratio prevent model overfitting by controlling model complexity. Given the class imbalance, the final model for each fold used the optimal hyperparameters maximizing validation macro-F1. The XGBoost model also inherently provides feature importance metrics. Ultimately, the XGBoost pipeline yields an ensemble of decision-tree models that predict bradykinesia task scores (i.e., pronation-supination score and toe tapping score) from predefined signal features, producing a single prediction per task segment from the concatenated window features.

**Table 1:** XGBoost hyperparameters and associated search ranges.

Hyperparameter	Description	Values
Colsample by tree	Fraction of features to sample for each tree	[0.6, 0.8]
Learning rate	Boosting learning rate; lower values tend to yield better test results but require more trees	[0.1, 0.3]
Max depth	Maximum depth of each tree	[4, 6, 8]
Number of estimators	Number of trees in the ensemble	[50, 100, 150]
Subsample	Fraction of samples to be used for each tree; prevents overfitting	[0.8, 1.0]

### 2.5 CNN model

**Figure 1:**
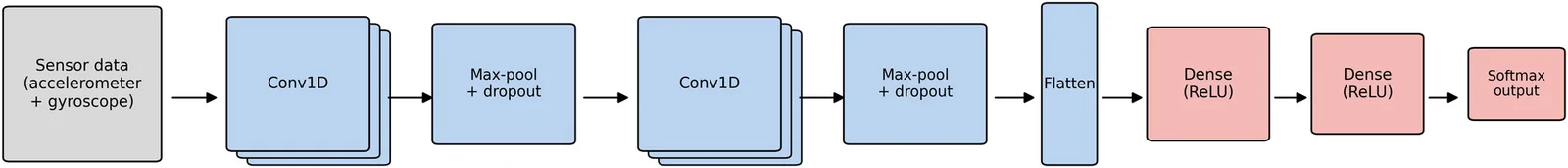
CNN model architecture. The raw multi-channel sensor time series (tri-axial accelerometer and gyroscope) passes through two convolutional blocks, each a 1D convolutional layer followed by max-pooling and dropout, then a flatten layer, two fully-connected (dense, ReLU) layers, and a softmax output over the four severity classes. The model uses only the sensor signals; no demographic inputs are included. Layer dimensions and the tuned kernel size are given in the Methods and Table 2. This figure was generated with the assistance of Claude Opus 4.8 and subsequently validated by the authors.

For the deep learning approach, we developed a 1D CNN model that operates directly on the raw sensor time-series. Each input to the CNN model was a 10 s sequence of multi-channel inertial data corresponding to one task performance. We included six sensor signal channels per sample (tri-axial accelerometer + gyroscope from the wrist or foot sensor on the limb performing the task), reflecting the multi-axis motion during pronation–supination or toe tapping. The raw sensor time-series input passed through two successive convolutional blocks (with 16 and 8 filters, respectively), each consisting of a 1D convolutional layer followed by a max-pooling layer and a dropout layer for regularization. The output from the two blocks was then flattened and passed through two fully-connected (dense) layers of sizes 16 and 8 (ReLU activations) to learn nonlinear combinations of the features. Finally, the network outputs a probability distribution over the four MDS-UPDRS score categories via a dense softmax layer, and the highest-probability class is taken as the predicted grade. The complete CNN architecture is illustrated in Figure 1.

**Table 2:** CNN Hyperparameters and associated search range.

Hyperparameter	Range
Batch size	16, 32
Kernel size	4, 8, 16, 32, 64
Drop out	0, 0.2, 0.4

The CNN was trained using a categorical cross-entropy loss function and optimized with RMSprop, a standard adaptive optimizer used as a sensible default that provided stable convergence for this compact architecture; for datasets and models of this size, the specific choice of adaptive optimizer is generally expected to have limited impact on final performance. We tuned the convolutional kernel size, dropout rate, and batch size by selecting the configuration with the best validation macro-F1 within the training folds (search ranges in Table 2). Informed by earlier coarse-to-fine experiments that favored smaller kernels for this high-frequency signal, the final search covered kernel sizes {4, 8, 16, 32, 64}, dropout rates {0, 0.2, 0.4}, and batch sizes {16, 32}. Additionally, we utilized an early stopping criterion on validation loss to prevent overfitting. We also experimented with L1 and L2 regularization techniques to enhance model generalization. However, we observed that the inclusion of regularizers did not improve model performance compared to the baseline configuration. Consequently, regularization was excluded from our final model training. Unlike the XGBoost model, the CNN learns its own feature representations directly from the raw signals, without manual feature engineering [15].

To address class imbalance and to test architectural sensitivity, we additionally trained two CNN variants on the same inputs and subject-level splits. First, a focal-loss variant replaced the categorical cross-entropy objective with a focal loss (γ=2, with inverse-frequency class weighting) to down-weight easy majority-class examples and emphasize hard minority-class cases. Second, a self-attention variant inserted a transformer-style multi-head self-attention block (with a residual connection and layer normalization) followed by global average pooling in place of the flatten operation, allowing the model to learn which time steps are most informative. Both variants were trained and selected under the same protocol as the base CNN.

### 2.6 Model training and evaluation

We adopted a consistent participant-wise cross-validation strategy to train and evaluate both the XGBoost and CNN models [16]. The full dataset was partitioned into five folds by subject, ensuring that all data from any given participant were contained within a single fold. This approach prevented any data from the same individual appearing in both training and test sets, thus avoiding optimistic bias due to within-subject correlations. Model training proceeded in five iterations of cross-validation: in each fold, 4/5 of the participants (and their corresponding task data) were used for training and the remaining 1/5 for testing. Performance metrics were computed on each fold’s held-out set and then aggregated across folds to obtain an overall evaluation. This yields predictions for the entire cohort (each participant appears once in a test fold) and provides a robust assessment of generalization. Both modeling approaches aimed to predict the discrete MDS-UPDRS task scores (0–3) as a classification problem. During training, hyperparameters for each model were tuned via internal cross-validation on the training folds. After training, the models’ predictions on each task trial were compared to the clinician-rated ground truth to evaluate overall accuracy and the models’ ability to distinguish different bradykinesia severity levels. As a lower-bound reference, we also evaluated a stratified-random baseline that predicts classes at random in proportion to the training-set label frequencies. Given the class imbalance, all tuned models were selected on validation macro-F1, and we report accuracy, balanced accuracy, macro-F1, quadratic weighted kappa, and per-class F1 on the held-out test folds, together with confusion matrices. We further assessed how each model’s predictions relate to the clinician-rated severity grade while accounting for the multiple recordings contributed by each participant. For this analysis, each model’s output was summarized as a continuous composite score, the probability-weighted expected grade, $\hat{s}=\sum_{k=0}^{3}k\;p_{k}$, where $p_{k}$ is the model’s predicted probability of grade k, which yields a value in [0,3] that is more sensitive to within-subject change than the discrete class used for the classification metrics. We then fit a linear mixed-effects model regressing this composite score on the clinical grade with a random intercept per subject, reporting the slope and its 95% confidence interval.

Longitudinal sensitivity analysis was conducted using Cohen’s d effect size between baseline and 12 months for the ML composite score. For comparison, we also conducted the same analysis for the previously established heuristic composite score by Czech et al. [13]. The heuristic composite score is a separate, signal-feature-based score not derived from the ML model outputs. Additionally, linear mixed-effects models were used to evaluate temporal changes in algorithmic performance from baseline through 12 months. Each model’s output served as the dependent variable, with time (measured continuously at 0, 1, 3, 6, 9, and 12 months) as the primary fixed effect, and random intercepts included to capture variability between individual subjects.

## 3 Results

### 3.1 Data description

In total, we have 132 subjects. Among these, 82 are Parkinson’s disease patients and 50 are healthy volunteers. The demographic information of the 132 subjects is shown in Table 3.

**Table 3:** Demographic characteristics of the study population.

Characteristic	Category	Overall
*n*		132
Age, mean (SD)		66.6 (9.7)
Male, *n* (%)	0	68 (51.5)
	1	64 (48.5)
Race_white, *n* (%)	0	6 (4.5)
	1	126 (95.5)

Categorical variables are coded as: male (1 = male, 0 = female) and race_white (1 = White, 0 = non-White).

**Figure 2:**
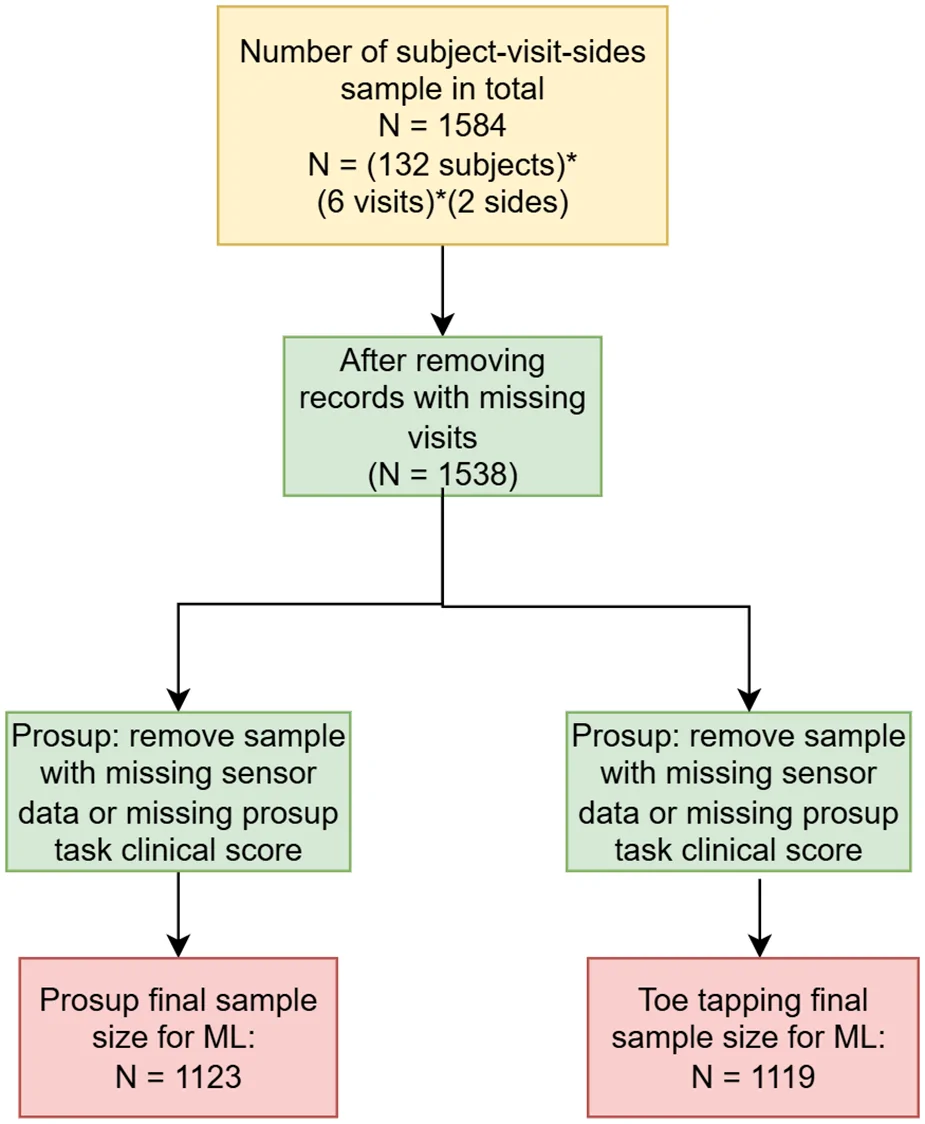
Analysis dataset sample size flow chart.

After removing missing data, we have 128 subjects, 1,123 data samples (subject-visit-side level) for pronation-supination task, and 128 subjects, 1,119 samples (subject-visit-side level) for the toe tapping task. For toe tapping, two recordings whose active-movement segment was too short (<4 s) to form the XGBoost windowed features were excluded from the XGBoost feature set; the XGBoost toe tapping analyses therefore use 1,117 recordings, whereas the CNN, which ingests the full segment, uses all 1,119. The process from raw data to the final analysis dataset is shown in Figure 2.

In the final analysis dataset, the distributions of the MDS-UPDRS scores for pronation-supination and toe tapping tasks are shown in Table 4.

**Table 4:** Distribution of pronation-supination and toe tapping scores by side.

MDS-UPDRS score	Pronation-supination score		Toe tapping score	
	Left	Right	Left	Right
0	295	304	264	299
1	141	154	169	146
2	85	72	84	83
≥3	43	29	41	33

### 3.2 Model selection and performance

The performance of the XGBoost and CNN models on the held-out test folds, including imbalance-aware metrics, is summarized in Table 5. For the pronation-supination task, XGBoost achieved an average test accuracy of 0.592 (SD 0.042) and macro-F1 of 0.420 (0.022), compared with the CNN (cross-entropy) at 0.528 (0.020) accuracy and 0.330 (0.027) macro-F1. For the toe tapping task, XGBoost reached 0.536 (0.069) accuracy and 0.398 (0.091) macro-F1, vs. 0.467 (0.025) accuracy and 0.275 (0.049) macro-F1 for the CNN. Across both tasks and across all imbalance-aware metrics (balanced accuracy, macro-F1, and quadratic weighted kappa), XGBoost outperformed the CNN, and every model substantially exceeded the stratified-random baseline (e.g., pronation-supination macro-F1 0.234 (0.022) with a near-zero quadratic weighted kappa).

**Table 5:** Imbalance-aware performance of the models on the held-out test set (mean (SD) over 5 subject-level repetitions, where the five folds partition participants so that all data from any participant fall in a single fold): a stratified-random baseline; CNNs (cross-entropy, focal loss, and self-attention); and XGBoost, both without and with class-balanced sample weighting.

Task	Model	Accuracy	Balanced Acc.	Macro-F1	QWK	F1_0_	F1_1_	F1_2_	F1_3_
Prosup	Stratified random	0.367 (0.024)	0.239 (0.025)	0.234 (0.022)	−0.017 (0.054)	0.524	0.255	0.145	0.013
	CNN (CE)	0.528 (0.020)	0.351 (0.021)	0.330 (0.027)	0.422 (0.076)	0.713	0.249	0.326	0.031
	CNN (focal)	0.369 (0.075)	0.352 (0.051)	0.298 (0.045)	0.286 (0.077)	0.530	0.266	0.167	0.228
	CNN (attention)	0.514 (0.032)	0.354 (0.069)	0.308 (0.069)	0.314 (0.138)	0.691	0.128	0.260	0.154
	XGBoost	0.592 (0.042)	0.417 (0.029)	0.420 (0.022)	0.526 (0.066)	**0.770**	0.328	0.265	0.318
	XGBoost (balanced)	**0.603 (0.044)**	**0.468 (0.048)**	**0.471 (0.033)**	**0.579 (0.071)**	0.768	**0.364**	**0.368**	**0.385**
Toetap	Stratified random	0.355 (0.031)	0.235 (0.040)	0.235 (0.040)	−0.011 (0.075)	0.502	0.272	0.138	0.029
	CNN (CE)	0.467 (0.025)	0.296 (0.046)	0.275 (0.049)	0.223 (0.138)	0.646	0.302	0.150	0.000
	CNN (focal)	0.373 (0.043)	0.358 (0.053)	0.316 (0.036)	0.253 (0.108)	0.505	0.338	0.210	0.210
	CNN (attention)	0.488 (0.043)	0.391 (0.055)	0.360 (0.069)	0.405 (0.080)	0.674	0.292	0.186	0.287
	XGBoost	**0.536 (0.069)**	0.412 (0.079)	0.398 (0.091)	0.461 (0.065)	**0.706**	0.320	**0.241**	0.325
	XGBoost (balanced)	0.526 (0.062)	**0.422 (0.068)**	**0.403 (0.072)**	**0.467 (0.095)**	0.692	**0.339**	0.237	**0.343**

All models were selected on validation macro-F1. F1_0_–F1_3_ are per-class F1 (class-wise means). Bold indicates the best value for each metric within each task.

The class-balanced variants further improved detection of the rare, more-severe grades. The class-balanced XGBoost gave the best overall performance: for pronation-supination it achieved the best performance on most metrics [accuracy 0.603 (0.044), balanced accuracy 0.468 (0.048), macro-F1 0.471 (0.033), and QWK 0.579 (0.071)], with substantially higher minority-class F1 than the unweighted XGBoost (class-2 F1 0.368 vs. 0.265; class-3 F1 0.385 vs. 0.318), and for toe tapping it likewise gave the best macro-F1 [0.403 (0.072)] and QWK [0.467 (0.095)]. For the CNN, focal loss raised the most-severe-class (score 3) F1 from 0.031 to 0.228 for pronation-supination and from 0.000 to 0.210 for toe tapping, at some cost to overall accuracy (0.369 (0.075) and 0.373 (0.043), vs. 0.528 (0.020) and 0.467 (0.025) for the cross-entropy CNN); the self-attention CNN gave the best CNN macro-F1 for toe tapping [0.360 (0.069)] and a comparable macro-F1 for pronation-supination [0.308 (0.069)]. Overall, the class-balanced XGBoost provided the best balance between overall accuracy and sensitivity to the minority classes. We deliberately retained the rare but clinically important severe grades (scores 2 and 3), accepting the resulting trade-off between overall accuracy and minority-class sensitivity; the corresponding confusion matrices are shown in Supplementary Figures S1–S4. The subsequent analyses were based on the best-performing model for each task: the class-balanced XGBoost, and the CNN (cross-entropy for pronation-supination and self-attention for toe tapping).

**Figure 3:**
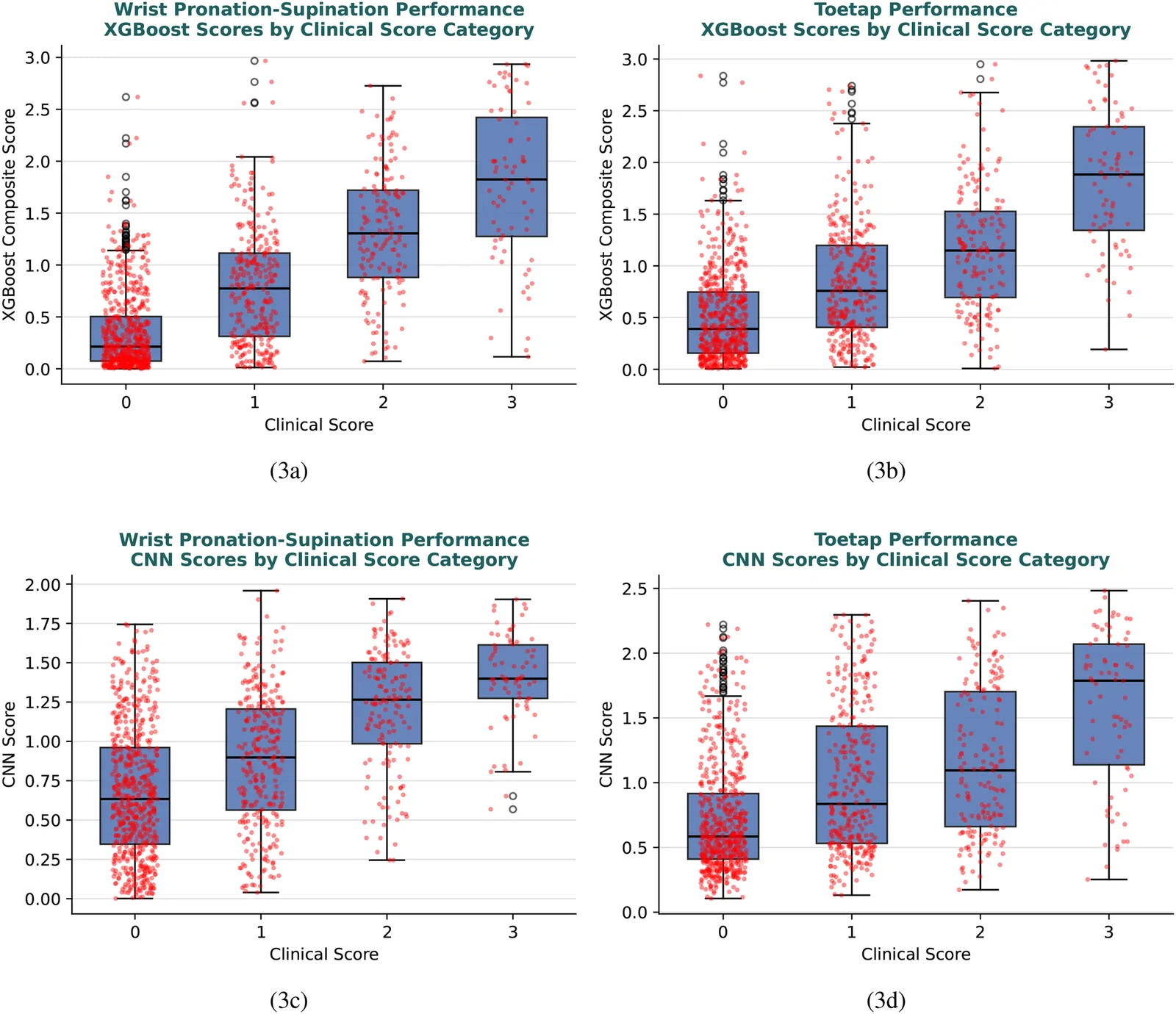
Boxplot distributions of model predictions by MDS-UPDRS clinical score categories. Predictions are the probability-weighted composite scores ($\sum_{k}k\;p_{k}$) of the best models: the class-balanced XGBoost, and the CNN (cross-entropy for pronation-supination; self-attention for toe tapping). Box plots display median (center line), interquartile range (IQR), and whiskers extending to the furthest points within 1.5 × IQR from the box edges. Individual data points are overlaid as red dots, with outliers shown beyond the whiskers. **(a,c)** XGBoost and CNN model predictions for pronation-supination task vs. MDS-UPDRS clinical score. The sample distribution across classes is: 599 instances for class 0, 295 instances for class 1, 157 instances for class 2, and 72 instances for class 3. **(b,d)** XGBoost and CNN model predictions for toe tapping task vs. MDS-UPDRS clinical score. The sample distribution across classes is: 563 for class 0, 315 for class 1, 167 for class 2, and 74 for class 3; the XGBoost toe tapping panel **(b)** excludes two class-0 recordings whose active segment was too short (<4 s) to form the windowed features (n=1,117; class-0 = 561).

To assess whether the model-predicted composite scores are associated with the clinician-rated severity grade, we fit a linear mixed-effects model in which the composite score is regressed on the clinical grade with a random intercept per subject (Figure 3). Predicted composite scores increased significantly with clinical grade for both tasks. For XGBoost, each one-grade increase in the clinician rating corresponded to a 0.42 (95% CI [0.39,0.46]) increase in composite score for pronation-supination and 0.36 ([0.32,0.40]) for toe tapping; the CNN showed positive but shallower slopes (0.20 [0.17,0.23] and 0.22 [0.19,0.26], respectively). All slopes were highly significant ($p<10^{-30}$).

### 3.3 Model sensitivity evaluation compared to heuristic approach

**Figure 4:**
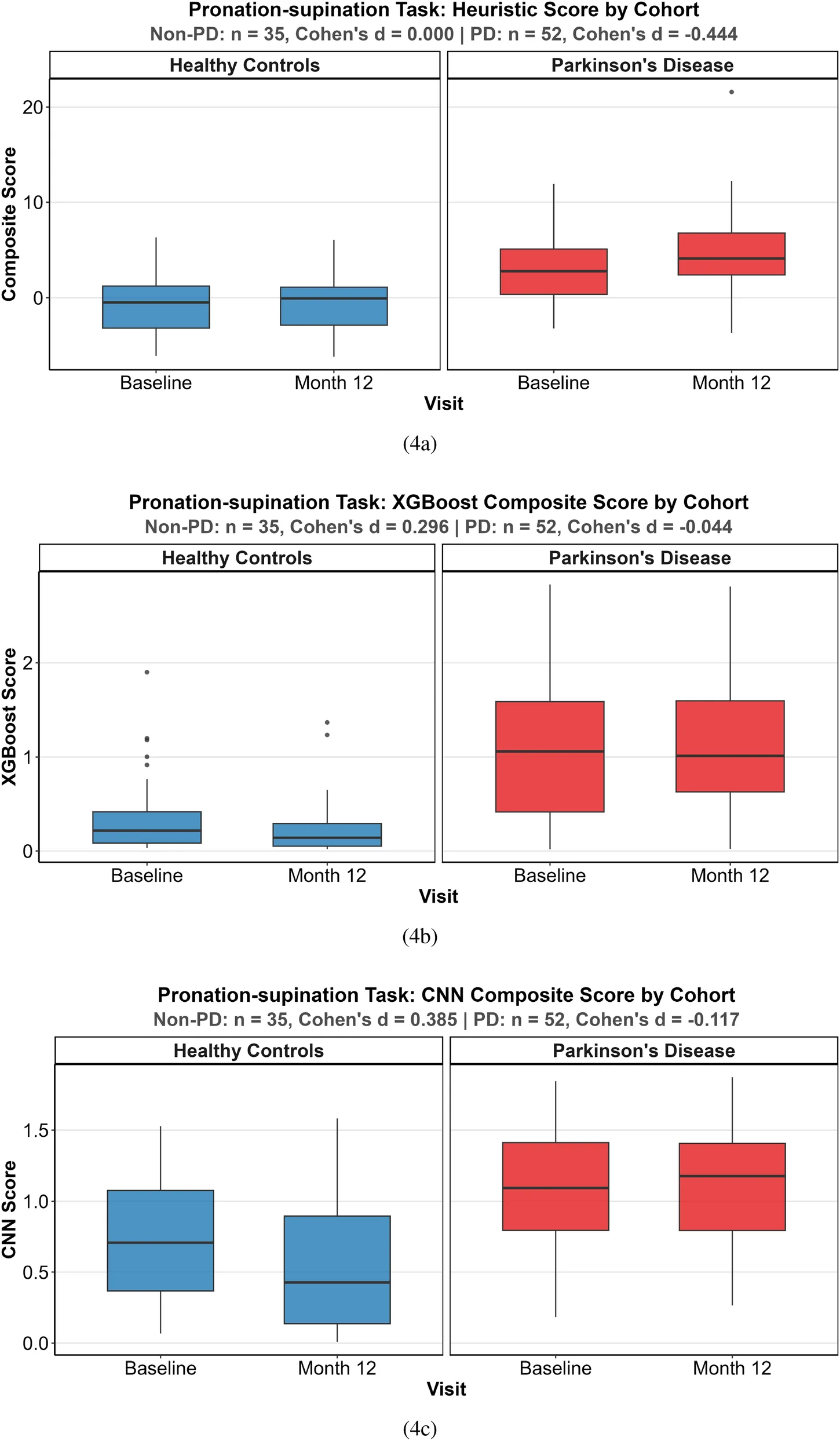
Box plots for pronation-supination display score distributions at baseline and 12-month follow-up for healthy controls (blue) and Parkinson’s disease patients (red). Box plots display median (center line), interquartile range (IQR), and whiskers extending to the furthest points within 1.5 × IQR from the box edges. **(a)** Heuristic score by cohort. **(b)** XGBoost model prediction score by cohort. **(c)** CNN prediction score by cohort. Paired Cohen’s d effect sizes were calculated to evaluate the within group (e.g., Parkinson’s disease) changes in model scores from baseline to 12-month follow-up. Only subjects that have both baseline visit data and 12-month data were included in the analysis.

**Figure 5:**
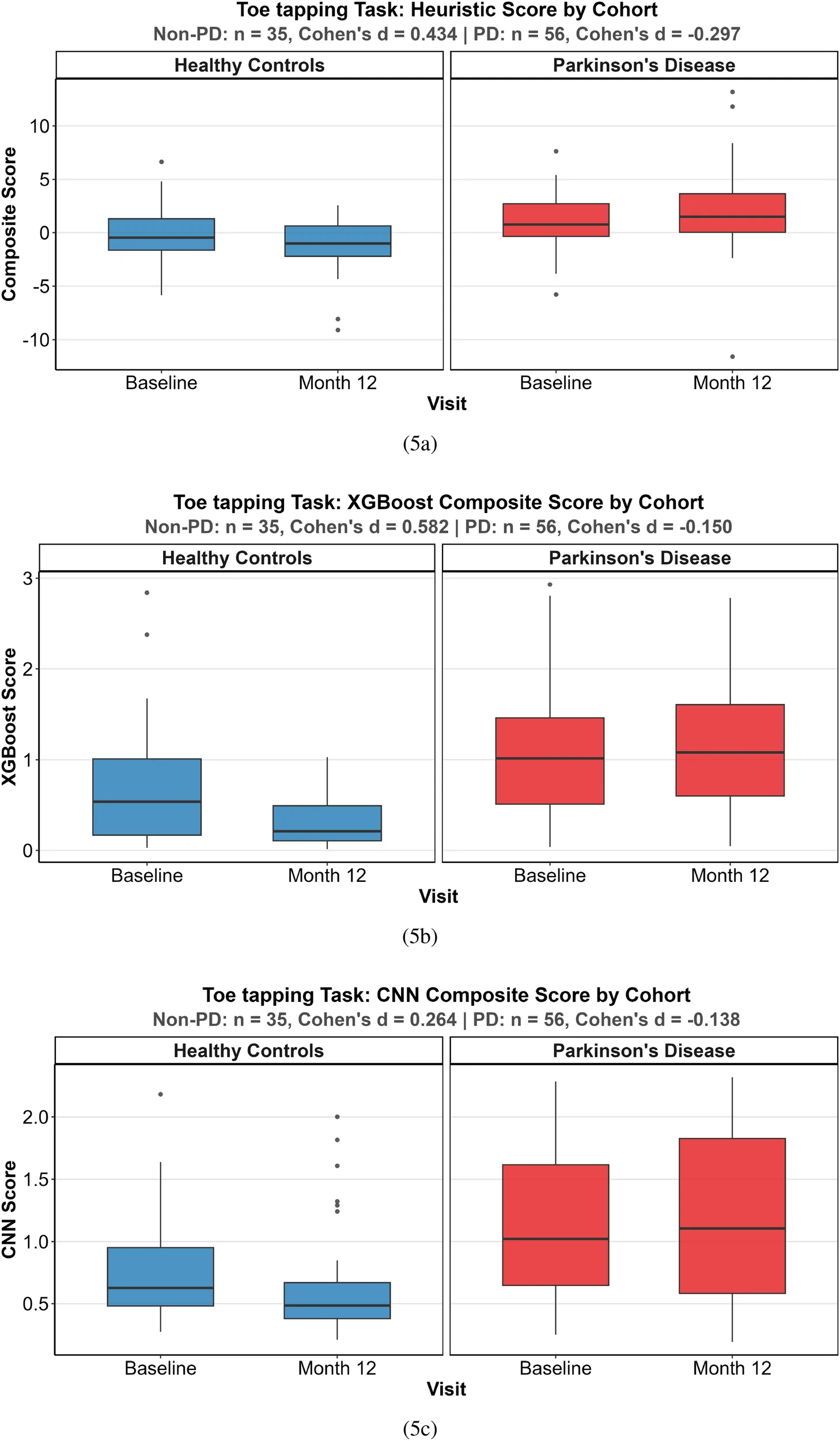
Box plots for toe tapping display score distributions at baseline and 12-month follow-up for healthy controls (blue) and Parkinson’s disease patients (red). Box plots display median (center line), interquartile range (IQR), and whiskers extending to the furthest points within 1.5 × IQR from the box edges. **(a)** Heuristic score by cohort. **(b)** XGBoost model prediction score by cohort. **(c)** CNN prediction score by cohort. Paired Cohen’s d effect sizes were calculated to evaluate the within group (e.g., Parkinson’s disease) changes in model scores from baseline to 12-month follow-up. Only subjects that have both baseline visit data and 12-month data were included in the analysis.

To assess the models’ ability to monitor disease progression in Parkinson’s disease patients, we calculated Cohen’s d effect sizes comparing baseline and 12-month visits across different scoring methods. Here Cohen’s d is computed as baseline minus 12-month, so a negative value means the score increased from baseline to month 12 (i.e., symptoms worsened), while the magnitude reflects the size of that change. For the pronation-supination task, among Parkinson’s disease patients, the heuristic composite score from Czech et al. [13] yielded a Cohen’s d of −0.444 (CI = [−0.808, −0.081]), indicating a moderate effect comparing baseline vs. 12-month visits, while our scores from the CNN and XGBoost models showed Cohen’s d values of −0.117 (CI = [−0.427, 0.194]) and −0.044 (CI = [−0.362, 0.275]), respectively, indicating minimal sensitivity to change. For the toe tapping task, the heuristic composite score by Czech et al. [13] demonstrated a Cohen’s d of −0.297 (CI = [−0.539, −0.055]), representing a small-to-moderate effect in detecting progression over the 12-month period. In contrast, the CNN and XGBoost models produced Cohen’s d values of −0.138 (CI = [−0.401, 0.124]) and −0.150 (CI = [−0.421, 0.122]), respectively, both reflecting negligible change detection. Notably, MDS-UPDRS pronation-supination and toe tapping item scores also showed minimal change over the 12-month period [13]. Detailed results of Cohen’s d tests for both healthy controls and PD cohort are presented in Figures 4, 5.

We also assessed the sensitivity of scores in detecting changes between baseline and 12-month visits among healthy controls, where little change would be expected. For the pronation-supination task, the heuristic approach demonstrated no detectable change from baseline to 12-month follow-up (baseline minus 12-month, Cohen’s d = 0.000, CI = [−0.270, 0.271]). The XGBoost and CNN scores gave small-to-moderate point estimates (XGBoost Cohen’s d = 0.296, CI = [−0.071, 0.663]; CNN Cohen’s d = 0.385, CI = [0.005, 0.765]), although the XGBoost estimate is not statistically significant. For the toe tapping task, the heuristic composite score and the XGBoost model showed small-to-moderate decreases (Cohen’s d = 0.434, CI = [0.067, 0.801] for heuristic; 0.582, CI = [0.235, 0.929] for XGBoost), whereas the CNN point estimate was smaller and not statistically significant (Cohen’s d = 0.264, CI = [−0.201, 0.729]).

We additionally examined the models’ sensitivity to progressive motor decline via linear mixed-effects regression analyses of longitudinal performance trajectories from baseline through 12-month follow-up. Comparing the results, we see heuristic score mixed effect model coefficients for pronation-supination (β=0.096, 95% CI = [0.032, 0.161], *p* = 0.003) and toe tapping (β=0.042, 95% CI = [−0.017, 0.100], *p* = 0.16) show positive trends in the Parkinson’s disease population over the 12 month period. In contrast, XGBoost model coefficients for pronation-supination (β=0.005, 95% CI = [−0.008, 0.017], *p* = 0.45) and toe tapping (β=0.007, 95% CI = [−0.005, 0.019], *p* = 0.23) show negligible change. Similarly, CNN model coefficients for pronation-supination (β=0.005, 95% CI = [−0.003, 0.011], *p* = 0.20) and toe tapping (β=0.003, 95% CI = [−0.007, 0.014], *p* = 0.51) show negligible change. Of note, MDS-UPDRS pronation-supination (β=0.010, 95% CI = [−0.005, 0.025], *p* = 0.19) and toe tapping (β=0.011, 95% CI = [−0.004, 0.026], *p* = 0.16) scores also do not demonstrate meaningful change over the 12 month period, indicating limited sensitivity to detect progression over this time frame.

## 4 Discussion

This study evaluated a range of modeling approaches, including a stratified-random baseline, XGBoost, and CNN, for predicting clinician-rated MDS-UPDRS bradykinesia item scores (pronation-supination and toe tapping) from wearable movement data. Both the feature-based and deep-learning models mapped movement data to clinical item scores with promising performance; across imbalance-aware metrics (macro-F1, balanced accuracy, and QWK), the class-balanced XGBoost achieved the best overall performance, outperforming the CNN variants and the unweighted XGBoost model. A secondary longitudinal analysis was less favorable for the ML models: the heuristic composite score was more sensitive to 12-month change, indicating that strong cross-sectional prediction does not by itself guarantee progression sensitivity.

The diversity in modeling approaches highlights the importance of feature selection tailored to specific model architectures. For instance, features specific to the heuristic model, such as change in amplitude and velocity over duration of task, were pivotal for its sensitivity to progression [13]. In contrast, the XGBoost model’s top-performing features (gyroscope magnitude coefficient of variation and standard deviation, frequency domain components) successfully aligned with clinical scores but were not necessarily predictive of progression. This underscores the necessity of aligning the model’s end goal with the feature selection process in digital health technology (DHT) based outcome models. Notably, the clinician-rated MDS-UPDRS scores themselves changed little over the 12-month window in this early cohort; because the models are trained to reproduce these scores, limited sensitivity to change is expected, and longer follow-up capturing greater cumulative progression may reveal stronger longitudinal sensitivity for the trained models.

The lower performance of CNNs may reflect both architectural and data-related constraints. Specifically, the dataset’s modest size and pronounced class imbalance likely limited CNN generalization and would predispose heavier sequence models (e.g., LSTM/GRU, temporal CNNs with residuals) to overfitting and poor minority-class performance [17, 18]. To test whether temporal-localization mechanisms help the CNN focus on diagnostically relevant bursts [19], we evaluated a self-attention variant. It provided only modest gains, producing the best CNN macro-F1 for toe tapping (0.360) and comparable performance for pronation-supination, but did not surpass the feature-based XGBoost, suggesting that at the present dataset size and class imbalance, attention alone does not close the gap. This is relevant in PD motor assessments, where brief high-amplitude or rhythm-disruptive segments may be more diagnostically meaningful than the overall signal average. Additionally, pronounced label imbalance (with most assessments scored 0, Table 4) likely suppressed minority-class performance and depressed overall accuracy, as reflected by low recall/F1 for classes 2–3 in several folds, even when applying class-balancing strategies. Despite these challenges, CNNs present an advantageous option for continuous passive monitoring, such as continuous electrocardiogram monitoring for arrhythmia detection, and extended active tasks such as the six-minute walk test (6MWT) [20–22]. Supporting literature suggests that deep learning models such as CNNs may outperform heuristic models in these contexts [10]. Conversely, for shorter tasks, heuristic models, which leverage specific signal features, may offer a more suitable alternative [7].

Beyond architectural considerations, practical deployment in clinical contexts requires attention to several factors. XGBoost requires segmented and summarized input features to capture temporal patterns, whereas CNNs can learn these patterns directly from raw full-length sequences due to their convolutional architecture. Computationally, all approaches are lightweight, with their main cost being the hyperparameter search. ML models trained end-to-end on raw signals may be more advantageous for long, continuous monitoring periods, such as home-based monitoring, though they require large datasets to optimize. In either case, choice of model must factor in anticipated endpoint (alignment vs. progression), data requirements, and interpretability.

The overarching goal remains to ensure model-derived data reflect both clinician observations and patient experience. Disentangling functional-performance features from patient-centric measures could elucidate which digital biomarkers are most relevant for treatment-effect detection, daily function, and progression monitoring [23]. Future multimodal frameworks combining multiple data modalities (sensor, clinical, imaging, patient reported measures) may yield more robust performance and greater generalizability across clinical and real-world settings. Moreover, incorporating patient-reported outcomes (e.g., symptom diaries, fatigue scales, activities of daily life (ADL) questionnaires) as auxiliary labels or multi-task learning targets could help bridge the gap between digital measures and patient-centric endpoints. This strategy aligns with regulatory guidance emphasizing use of digital endpoints that are not only valid and reliable but also meaningful to patients [24]. Investigating these goals across larger, more diverse studies will help demonstrate generalizability, as the current study was limited to ePD participants, relied only on in-clinic data, and included only bradykinesia-related assessments. Additional limitations include that we did not model symptomatic medication initiation during follow-up (a potential confounder of progression), did not collect repeated independent clinician ratings to benchmark model agreement against inter-rater variability, and did not establish a minimal clinically important difference for these single-item, sensor-derived scores; we identify these as directions for future work.

## 5 Conclusions

In conclusion, in this study we developed a variety of XGBoost and CNN models to predict MDS-UPDRS severity scores for the pronation-supination and toe tapping tasks. Our results show that ML models hold promise for accurately predicting these outcomes. Nevertheless, our model results show limited sensitivity to longitudinal progression, emphasizing the need for models aligned with specific objectives. With larger datasets, multimodal data fusion, and advanced ML techniques, the accuracy of these item-level MDS-UPDRS predictions can be further improved, supporting their potential as objective, sensor-based measures of bradykinesia severity.

## Data Availability

The data that support the findings of this study are not openly available due to data privacy controls in place as part of the consortium that this work support, and all data are available to members of the Critical Path for Parkinson's Consortium 3DT Initiative Stage 2. For those who are not a part of 3DT Stage 2, a proposal may be made to the WATCH-PD Steering Committee (via the corresponding author) for de-identified baseline datasets.
