# Supplementary material for: Comparison of machine learning models for bradykinesia assessment using digital health technology
Source: Front Digit Health. 2026 Aug 21;8:1854818. doi: 10.3389/fdgth.2026.1854818 (PMC13543157; doi:10.3389/fdgth.2026.1854818)
Supplement: Supplementary file 1 [file Datasheet1.pdf]

Supplementary Material

1 SUPPLEMENTARY DATA

N/A

2 SUPPLEMENTARY TABLES AND FIGURES

2.1 Figures

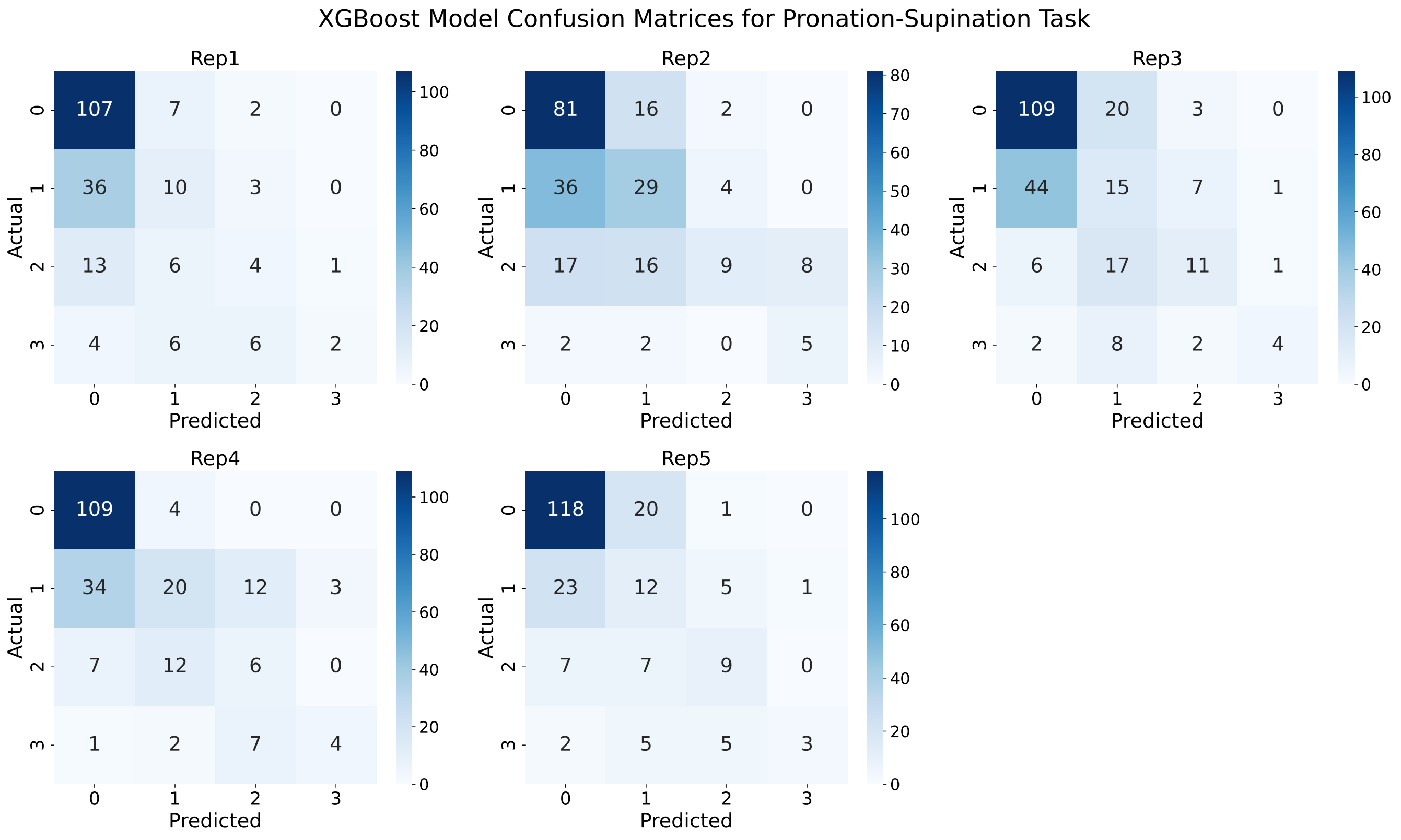

Figure S1. Performance evaluation of XGBoost model on pronation-supination task using confusion matrices across five folds (Rep1-Rep5). Each 4×4 matrix represents the classification performance for MDS-UPDRS scores ranging from 0 (normal) to 3 or 4 (moderate to severe impairment). Cell values indicate the number of samples, with perfect predictions along the main diagonal.

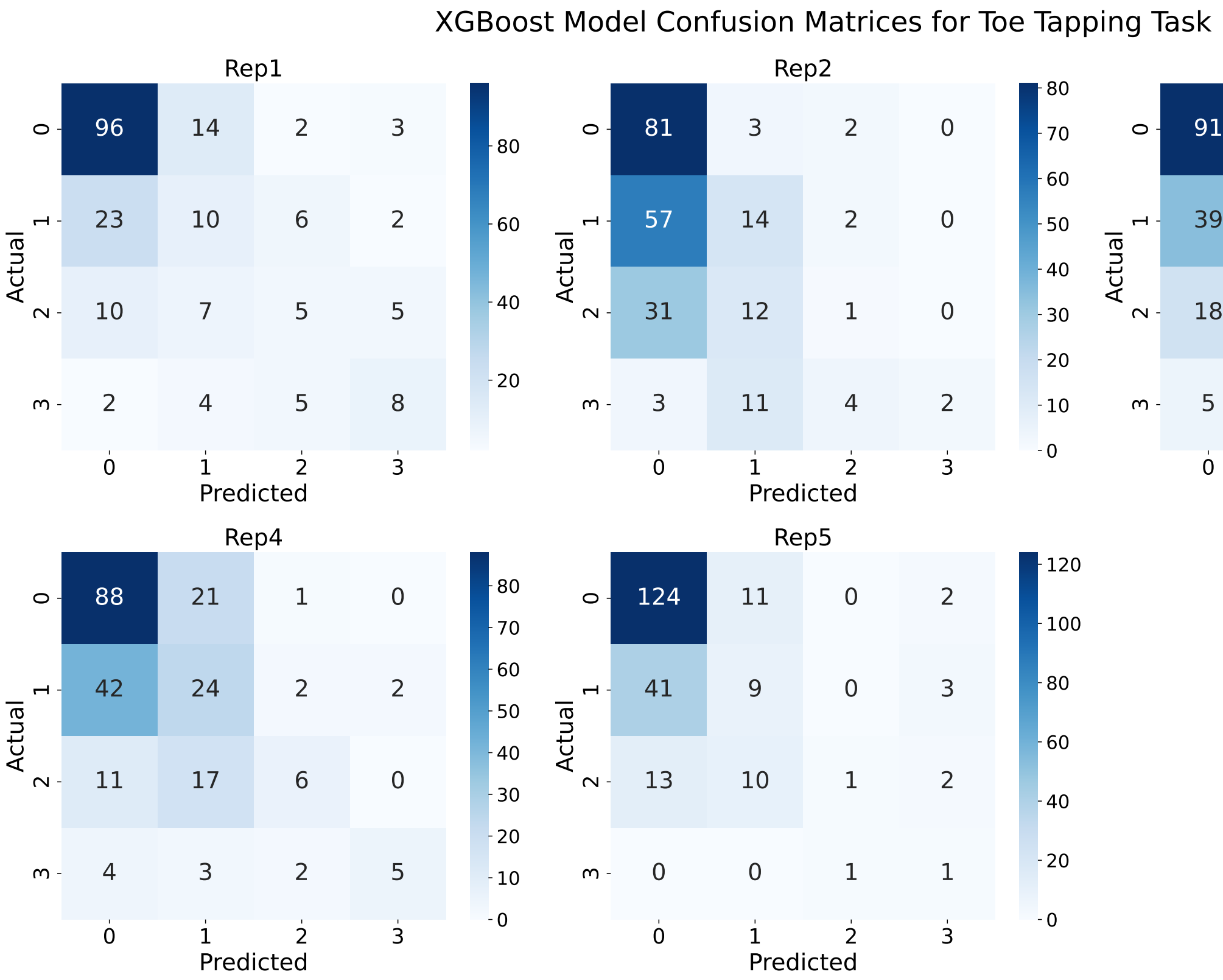

Figure S2. Performance evaluation of XGBoost model on toe tapping task using confusion matrices across five folds (Rep1-Rep5). Each 4×4 matrix represents the classification performance for MDS-UPDRS scores ranging from 0 (normal) to 3 or 4 (moderate to severe impairment). Cell values indicate the number of samples, with perfect predictions along the main diagonal.

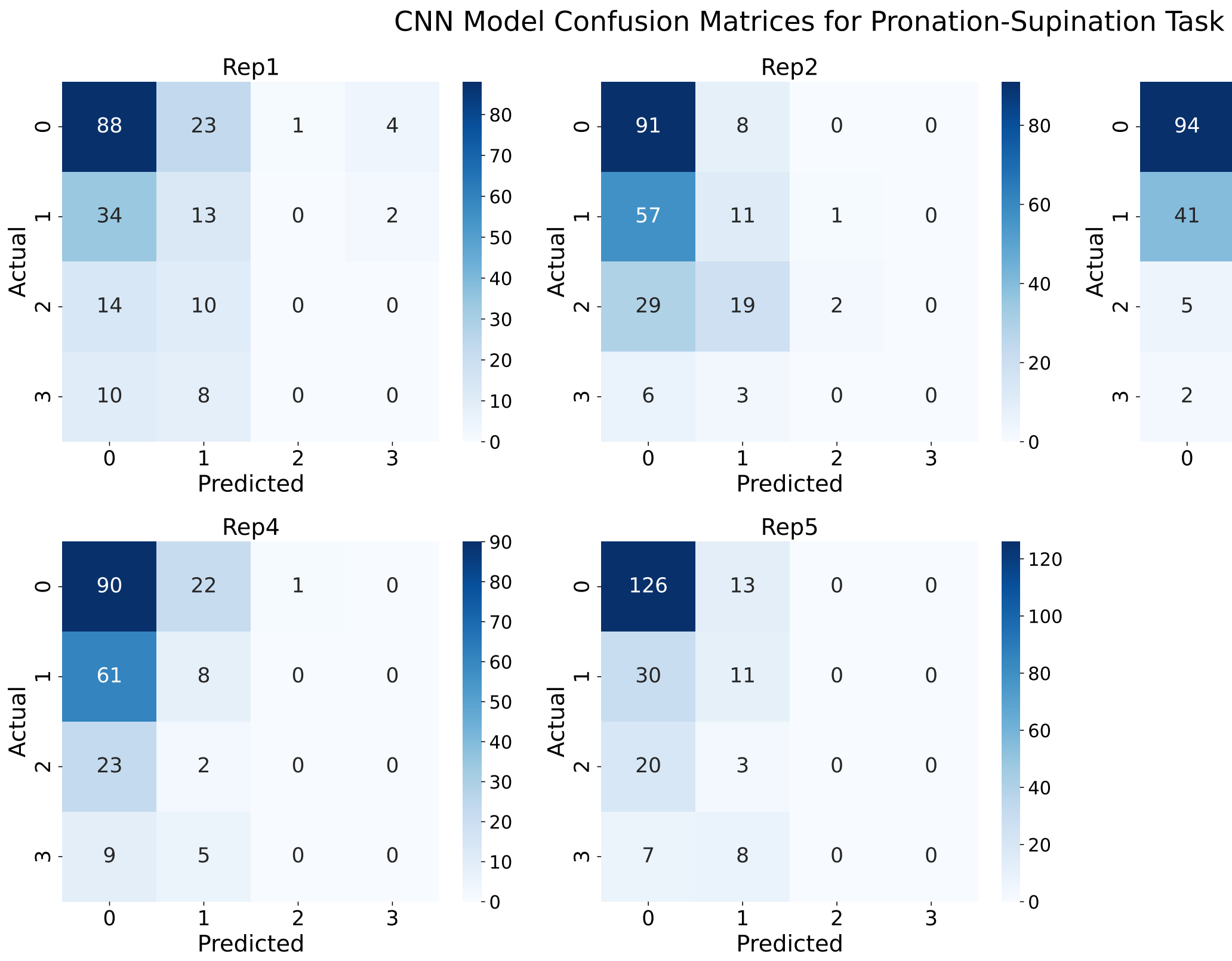

Figure S3. Performance evaluation of CNN model on pronation-supination task using confusion matrices across five folds (Rep1-Rep5). Each 4×4 matrix represents the classification performance for MDS-UPDRS scores ranging from 0 (normal) to 3 or 4 (moderate to severe impairment). Cell values indicate the number of samples, with perfect predictions along the main diagonal.

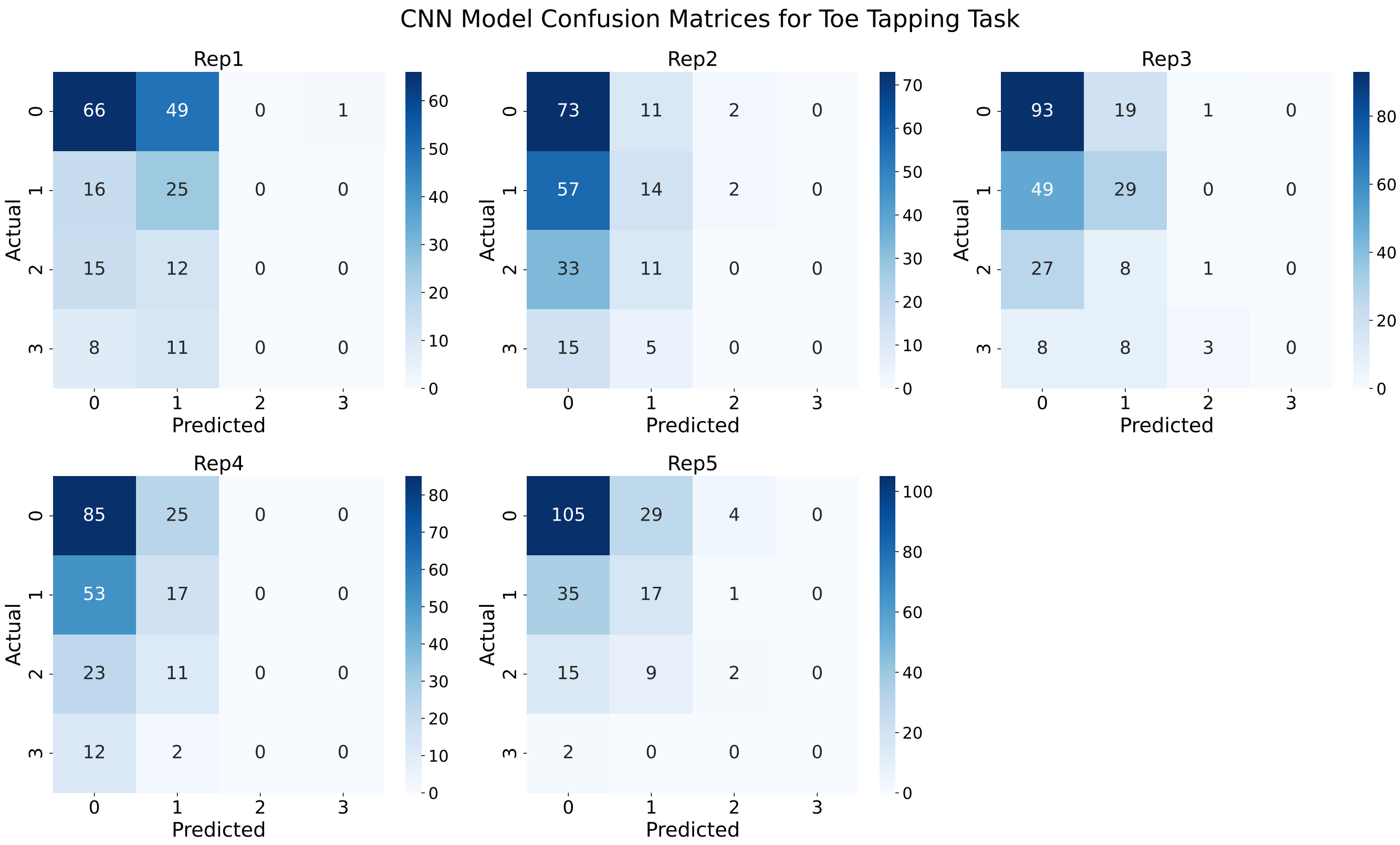

Figure S4. Performance evaluation of CNN model on toe tapping task using confusion matrices across five folds (Rep1-Rep5). Each 4×4 matrix represents the classification performance for MDS-UPDRS scores ranging from 0 (normal) to 3 or 4 (moderate to severe impairment). Cell values indicate the number of samples, with perfect predictions along the main diagonal.

2.2 Tables

Table S1. XGBoost Classification Performance Metrics by Task and Repetition

Task	Repeats	Class	Precision	Recall	F1-Score	Support
Prosup	Rep1	0	0.6688	0.9224	0.7754	116
		1	0.3448	0.2041	0.2564	49
		2	0.2667	0.1667	0.2051	24
		3	0.6667	0.1111	0.1905	18
	Rep2	0	0.5956	0.8182	0.6894	99
		1	0.4603	0.4203	0.4394	69
		2	0.6000	0.1800	0.2769	50
		3	0.3846	0.5556	0.4545	9
	Rep3	0	0.6770	0.8258	0.7440	132
		1	0.2500	0.2239	0.2362	67
		2	0.4783	0.3143	0.3793	35
		3	0.6667	0.2500	0.3636	16
	Rep4	0	0.7219	0.9646	0.8258	113
		1	0.5263	0.2899	0.3738	69
		2	0.2400	0.2400	0.2400	25
		3	0.5714	0.2857	0.3810	14
	Rep5	0	0.7867	0.8489	0.8166	139
		1	0.2727	0.2927	0.2824	41
		2	0.4500	0.3913	0.4186	23
		3	0.7500	0.2000	0.3158	15
Toetap	Rep1	0	0.7328	0.8348	0.7805	115
		1	0.2857	0.2439	0.2632	41
		2	0.2778	0.1852	0.2222	27
		3	0.4444	0.4211	0.4324	19
	Rep2	0	0.4709	0.9419	0.6279	86
		1	0.3500	0.1918	0.2478	73
		2	0.1111	0.0227	0.0377	44
		3	1.0000	0.1000	0.1818	20
	Rep3	0	0.5948	0.8053	0.6842	113
		1	0.4667	0.3590	0.4058	78
		2	0.2105	0.1111	0.1455	36
		3	0.5714	0.4211	0.4848	19
	Rep4	0	0.6069	0.8000	0.6902	110
		1	0.3692	0.3429	0.3556	70
		2	0.5455	0.1765	0.2667	34
		3	0.7143	0.3571	0.4762	14
	Rep5	0	0.6966	0.9051	0.7873	137
		1	0.3000	0.1698	0.2169	53
		2	0.5000	0.0385	0.0714	26
		3	0.1250	0.5000	0.2000	2

Table S2. CNN Classification Performance Metrics by Task and Repetition

Task	Repeats	Class	Precision	Recall	F1-Score	Support
Prosup	Rep1	0	0.6027	0.7586	0.6718	116
		1	0.2407	0.2653	0.2524	49
		2	0.0000	0.0000	0.0000	24
		3	0.0000	0.0000	0.0000	18
	Rep2	0	0.4973	0.9192	0.6454	99
		1	0.2683	0.1594	0.2000	69
		2	0.6667	0.0400	0.0755	50
		3	0.0000	0.0000	0.0000	9
	Rep3	0	0.6620	0.7121	0.6861	132
		1	0.2308	0.3134	0.2658	67
		2	0.1765	0.0857	0.1154	35
		3	0.0000	0.0000	0.0000	16
	Rep4	0	0.4918	0.7965	0.6081	113
		1	0.2162	0.1159	0.1509	69
		2	0.0000	0.0000	0.0000	25
		3	0.0000	0.0000	0.0000	14
	Rep5	0	0.6885	0.9065	0.7826	139
		1	0.3143	0.2683	0.2895	41
		2	0.0000	0.0000	0.0000	23
		3	0.0000	0.0000	0.0000	15
Toetap	Rep1	0	0.6286	0.5690	0.5973	116
		1	0.2577	0.6098	0.3623	41
		2	0.0000	0.0000	0.0000	27
		3	0.0000	0.0000	0.0000	19
	Rep2	0	0.4101	0.8488	0.5530	86
		1	0.3415	0.1918	0.2456	73
		2	0.0000	0.0000	0.0000	44
		3	0.0000	0.0000	0.0000	20
	Rep3	0	0.5254	0.8230	0.6414	113
		1	0.4531	0.3718	0.4085	78
		2	0.2000	0.0278	0.0488	36
		3	0.0000	0.0000	0.0000	19
	Rep4	0	0.4913	0.7727	0.6007	110
		1	0.3091	0.2429	0.2720	70
		2	0.0000	0.0000	0.0000	34
		3	0.0000	0.0000	0.0000	14
	Rep5	0	0.6688	0.7609	0.7119	138
		1	0.3091	0.3208	0.3148	53
		2	0.2857	0.0769	0.1212	26
		3	0.0000	0.0000	0.0000	2

Table S3. Model Performance (Macro F1) Comparison across Different Tasks and Repeats.

Task	Repeats	XGBoost Test	CNN Test
Prosup	Rep1	0.3569	0.2311
	Rep2	0.4651	0.2302
	Rep3	0.4308	0.2668
	Rep4	0.4552	0.1898
	Rep5	0.4584	0.2680
	**Average (SD)**	**0.4332 (0.0399)**	**0.2372 (0.0288)**
Toetap	Rep1	0.4246	0.2399
	Rep2	0.2738	0.1997
	Rep3	0.4301	0.2747
	Rep4	0.4472	0.2182
	Rep5	0.3189	0.2870
	**Average (SD)**	**0.3789 (0.0693)**	**0.2439 (0.0330)**

Table S4. Grouped accelerometer and gyroscope feature sets.

feature group	feature names
acc_x_percentiles	acc_x_min;acc_x_5th;acc_x_25th;acc_x_median;acc_x_75th;acc_x_95th; acc_x_max;acc_x_range
acc_y_percentiles	acc_y_min;acc_y_5th;acc_y_25th;acc_y_median;acc_y_75th;acc_y_95th; acc_y_max;acc_y_range
acc_z_percentiles	acc_z_min;acc_z_5th;acc_z_25th;acc_z_median;acc_z_75th;acc_z_95th; acc_z_max;acc_z_range
acc_norm_percentiles	acc_norm_min;acc_norm_5th;acc_norm_25th;acc_norm_median; acc_norm_75th;acc_norm_95th;acc_norm_max;acc_norm_range
acc_x_moments	acc_x_mean;acc_x_var;acc_x_std;acc_x_cov
acc_y_moments	acc_y_mean;acc_y_var;acc_y_std;acc_y_cov
acc_z_moments	acc_z_mean;acc_z_var;acc_z_std;acc_z_cov
acc_norm_moments	acc_norm_mean;acc_norm_var;acc_norm_std;acc_norm_cov
acc_x_shape	acc_x_skew;acc_x_kurt
acc_y_shape	acc_y_skew;acc_y_kurt
acc_z_shape	acc_z_skew;acc_z_kurt
acc_norm_shape	acc_norm_skew;acc_norm_kurt
acc_axis_correlations	acc_corr_xy;acc_corr_xz;acc_corr_yz
acc_norm_peak_freqs	acc_norm_f1;acc_norm_f2
acc_norm_peak_amps	acc_norm_p1;acc_norm_p2
acc_norm_power_metrics	acc_norm_totpwr;acc_norm_p1pct;acc_norm_p2pct;acc_norm_pwratio
acc_norm_spectral_entropy	acc_norm_entropy
gyro_x_percentiles	gyro_x_min;gyro_x_5th;gyro_x_25th;gyro_x_median;gyro_x_75th; gyro_x_95th;gyro_x_max;gyro_x_range
gyro_y_percentiles	gyro_y_min;gyro_y_5th;gyro_y_25th;gyro_y_median;gyro_y_75th; gyro_y_95th;gyro_y_max;gyro_y_range
gyro_z_percentiles	gyro_z_min;gyro_z_5th;gyro_z_25th;gyro_z_median;gyro_z_75th; gyro_z_95th;gyro_z_max;gyro_z_range
gyro_norm_percentiles	gyro_norm_min;gyro_norm_5th;gyro_norm_25th;gyro_norm_median; gyro_norm_75th;gyro_norm_95th;gyro_norm_max;gyro_norm_range
gyro_x_moments	gyro_x_mean;gyro_x_var;gyro_x_std;gyro_x_cov
gyro_y_moments	gyro_y_mean;gyro_y_var;gyro_y_std;gyro_y_cov
gyro_z_moments	gyro_z_mean;gyro_z_var;gyro_z_std;gyro_z_cov
gyro_norm_moments	gyro_norm_mean;gyro_norm_var;gyro_norm_std;gyro_norm_cov
gyro_x_shape	gyro_x_skew;gyro_x_kurt
gyro_y_shape	gyro_y_skew;gyro_y_kurt
gyro_z_shape	gyro_z_skew;gyro_z_kurt
gyro_norm_shape	gyro_norm_skew;gyro_norm_kurt
gyro_axis_correlations	gyro_corr_xy;gyro_corr_xz;gyro_corr_yz
gyro_norm_peak_freqs	gyro_norm_f1;gyro_norm_f2
gyro_norm_peak_amps	gyro_norm_p1;gyro_norm_p2
gyro_norm_power_metrics	gyro_norm_totpwr;gyro_norm_p1pct;gyro_norm_p2pct;gyro_norm_pwratio
gyro_norm_spectral_entropy	gyro_norm_entropy
